# From Molecular Cleavage to Clinical Effect: A Probabilistic Field Model of Botulinum Toxin Action

**DOI:** 10.3390/biology15050446

**Published:** 2026-03-09

**Authors:** Andrea Felice Armenti, Francesco Armenti

**Affiliations:** 1 LA VISION Training Institute, Via Magenta, 5, 00185 Rome, Italy; 2 Independent Researcher, 00185 Rome, Italy

**Keywords:** botulinum toxins, SNAP-25 protein, SNARE complex proteins, synaptic transmission, spatial distribution, molecular systems biology, presynaptic terminals

## Abstract

Botulinum toxin (BoNT) is a highly specific molecular enzyme whose therapeutic action is based on the proteolytic cleavage of SNARE proteins, most notably SNAP-25. Despite the deterministic nature of this molecular mechanism, the clinical effects of BoNT exhibit substantial variability in efficacy, spatial extent, and duration that cannot be fully explained by dose–response relationships or diffusion-based models. In this work, we propose the Molecular Probability Field (MPF-BoNT) as a conceptual framework that bridges discrete molecular events and emergent functional outcomes. The MPF is defined as the spatial–temporal distribution of the probability that presynaptic terminals reach a functional silencing state (operationalized via SNAP-25 cleavage exceeding a threshold), shaped by exposure, uptake, target density, and temporal dynamics following toxin exposure. Within this framework, clinical effects arise from the integration of probabilistic molecular events across space and time, rather than from toxin presence or concentration alone. The MPF-BoNT framework accounts for key features of botulinum toxin action, including spread, nonlinearity of dose effects, variability in duration, and differences between technical and biological non-response. By explicitly incorporating molecular variables such as local concentration, exposure time, terminal density, internalization probability, and functional silencing thresholds, the framework provides an integrative interpretation of tissue-level behavior grounded in molecular biology. The MPF-BoNT offers a formal language to describe how established enzymatic events generate observable spatial, temporal, and functional patterns. As a generative framework grounded in explicit testable structure, it establishes a foundation for future experimental and clinical research.

## Simple Summary

Botulinum toxin is widely used in medicine and aesthetics to reduce muscle activity, yet its clinical effects often vary even when the same dose is administered. Traditionally, these variations have been explained mainly in terms of how the drug spreads or how much is injected. However, this approach does not fully account for why similar doses can produce different spatial patterns of effect. In this study, we propose a new conceptual framework that describes treatment effects as a probability field across tissue. Instead of focusing only on drug concentration, the framework considers the likelihood that individual nerve endings become functionally silenced after exposure. This probability depends on several biological factors, including local exposure, cellular uptake, molecular activity inside the cell, and the threshold required to impair nerve signaling. The framework does not introduce new biological mechanisms but provides a structured way to connect molecular events to observable clinical outcomes across space and time. By clarifying how spatial patterns of response emerge, this approach may help guide future experimental research and improve the interpretation of variability in therapeutic and aesthetic applications.

## 1. Introduction

Botulinum neurotoxins (BoNTs) are zinc-dependent proteases produced by *Clostridium botulinum* that block neurotransmitter release by targeting proteins in the complex of the N-ethylmaleimide-sensitive factor attachment receptor (SNARE) [1]. Among the seven serotypes (A–G), BoNT/A is the most studied both in clinical and molecular contexts. Its remarkable specificity for the cleavage of the 25 kDa synaptosomal-associated protein (SNAP-25) underlies the reduction in acetylcholine release at the neuromuscular junction, a mechanism that is exploited therapeutically in conditions ranging from dystonia to spasticity and pain disorders [2,3].

Despite the well-characterized enzymatic action of BoNTs in synapses, there remains a conceptual gap in understanding how a binary molecular event—the proteolytic cleavage of a SNARE protein—gives rise to a continuum of functional outcomes in tissue and clinical settings. Empirical descriptors such as “spread’’ and “diffusion’’ are often invoked but lack formalization in a molecular systems framework [4].

We propose a generative probabilistic framework (MPF-BoNT) that defines a structural relationship between molecular events and spatial–temporal functional emergence. The present work formalizes this architecture without introducing empirical calibration or predictive parametrization. By anchoring the model in explicit molecular variables, we bridge the gap between well-established enzyme mechanics and emergent functional effects without invoking poorly defined phenomenological constructs.

## 2. Molecular Foundations of Botulinum Toxin Action

At the molecular level, BoNTs consist of a heavy chain responsible for receptor recognition and internalization and a light chain that acts as a metalloprotease targeting SNARE proteins [1,2]. The catalytic activity of the light chain cleaves SNAP-25 (for BoNT/A and BoNT/E) or other components of SNARE such as vesicle-associated membrane protein (VAMP) and syntaxin depending on the specificity of the serotype [2,5]. This cleavage prevents the formation of a functional SNARE complex, thus inhibiting synaptic vesicle fusion and acetylcholine release [6].

The enzymatic action of BoNT/A is highly specific and occurs only after receptor-mediated endocytosis into presynaptic terminals. Zinc-dependent proteolytic activity has been demonstrated in vitro with recombinant light chains and is sensitive to divalent cation chelators, confirming its catalytic nature [1]. Although the enzymatic mechanism is necessary to block neurotransmission, it is not sufficient to explain the full range of observed tissue-level effects. Differences in duration of action, onset kinetics, and functional spread cannot be predicted solely from enzymatic cleavage and require a system-level description that accounts for probabilistic uptake and spatial–temporal distribution of cleavage events.

This section establishes the molecular premises on which the subsequent probabilistic field model is constructed, explicitly linking the biochemical specificity of BoNT action to higher-order functional outcomes.

## 3. From Molecular Events to Tissue-Level Effects: The Conceptual Gap

### 3.1. Why Molecular Certainty Does Not Imply Clinical Determinism

At the molecular level, the action of botulinum toxin is remarkably well defined: receptor binding, internalization, and enzymatic cleavage of SNAP-25 constitute a deterministic biochemical sequence [1,2]. Nevertheless, the robustness of this molecular mechanism does not translate into deterministic tissue-level or clinical outcomes. Identical serotypes, doses, and target muscles may produce markedly different functional effects between individuals and anatomical contexts [4].

This apparent discrepancy reflects the fact that the elementary molecular event—proteolytic cleavage of SNAP-25—is binary and localized and occurs at the level of individual presynaptic terminals. In contrast, the observed functional outcome emerges from the collective behavior of a spatially distributed population of terminals. Therefore, clinical efficacy depends not solely on whether molecular cleavage occurs, but on how many terminals are affected, where they are located, and over what temporal window these events unfold.

Detailed structural and biochemical studies have demonstrated that botulinum toxin serotypes exhibit distinct substrate recognition profiles and multi-pocket interactions with SNAP-25, illustrating that substrate recognition can be structurally constrained and serotype-dependent, which motivates a probabilistic rather than strictly dose-deterministic linkage to tissue-level outcomes [7]. These serotype-dependent nuances further decouple molecular certainty from predictable functional outcomes.

Temporal dynamics introduce an additional layer of variability. The start and duration of the effect depend not only on the enzymatic activity but also on the internalization kinetics, the persistence of the active light chain, and compensatory synaptic mechanisms such as sprouting and functional reorganization [6]. As a result, deterministic molecular events give rise to probabilistic outcomes when integrated across space and time.

### 3.2. Current Implicit Models and Their Limitations

In the absence of a formal framework linking molecular events to tissue-level behavior, several implicit explanatory models dominate the literature. The most common invokes passive diffusion, implicitly assuming that the toxin molecules spread isotropically from the injection site until diluted below an effective threshold [8]. Although this description captures certain empirical observations, it overlooks the receptor-mediated and highly selective nature of botulinum toxin uptake and fails to distinguish the molecular presence from the enzymatic action.

A second widely assumed framework relies on a linear dose–response relationship, whereby increasing the injected dose is expected to proportionally increase clinical effect. However, saturation phenomena, nonlinear responses, and substantial inter-individual variability are consistently reported, indicating that dose alone is insufficient to predict functional outcomes [3]. Such dose-centric interpretations neglect the spatial organization of synaptic targets and the probabilistic nature of molecular interactions at the cellular level.

Finally, descriptive constructs such as “spread” or “field of effect” are frequently employed to account for the action of toxin beyond the injection site. Although these terms acknowledge the spatial extent of botulinum toxin effects, they remain qualitative and lack explicit molecular or spatial parameters. As a consequence, they offer limited explanatory or predictive power and cannot be readily translated into experimentally testable hypotheses.

Collectively, these limitations underscore the need for a formal model that explicitly links discrete molecular events to their spatial and temporal distribution in tissue, thus providing a principled explanation for the variability, spread, and duration of botulinum toxin effects.

## 4. The Molecular Probability Field Model (MPF-BoNT)

### 4.1. Definition of the Molecular Probability Field

We define the Molecular Probability Field (MPF) of botulinum toxin action as the spatial–temporal distribution of the probability that a presynaptic terminal undergoes sufficient SNAP-25 cleavage to exceed a functional silencing threshold following toxin exposure.

Let

(1) $$F\left(x,t\right)\equiv \left\{C_{\mathrm{SNAP}25}\left(x,t\right)\geq \theta \right\}$$

denote the terminal-level functional event. The MPF is then defined as

(2) $$\mathrm{MPF}\left(x,t\right)=P\;\left(F(x,t)\right).$$

A schematic probabilistic decomposition can be written without imposing independence assumptions by conditioning on elementary terminal-level events. Let *I* denote the event of productive internalization and *K* the event of catalytic competence over the relevant time window. Then

(3) $$\mathrm{MPF}\left(x,t\right)=\sum_{i\in \{0,1\}}\sum_{k\in \{0,1\}}P\;\left(F|I=i,K=k\right)P\;\left(K=k|I=i\right)P\;\left(I=i|x,t\right).$$

Supplementary Material S1 provides the corresponding formal development and interpretations.

Equations (2) and (3) fully define the structural object of the MPF within the main manuscript; Supplementary Material S1 expands their mathematical implications without introducing additional model components. This decomposition is conceptual rather than fully parameterized, but makes explicit the multilevel structure underlying the MPF object.

Clarification of scope. Although Equation (2) is written in terms of SNAP-25 cleavage, the MPF does *not* model cleavage probability in isolation. Rather, Equation (2) defines a functional event at the terminal-level (exceeding a silencing threshold) whose probability is determined by upstream determinants, including local exposure, receptor binding and internalization, catalytic efficiency, target density, and time. In other words, cleavage is the molecular readout, while the MPF is the integrative probabilistic object that links molecular engagement to functional silencing across space and time.

Here, $C_{\mathrm{SNAP}25}\left(x,t\right)$ denotes a random variable capturing the local fraction of SNAP-25 molecules cleaved within presynaptic terminals at spatial location *x* and time *t*, and θ denotes a threshold level of functional silencing. In this formulation, θ is treated as a (possibly distributed) functional criterion rather than as an additional stochastic event.

Biologically, θ should not be interpreted as a fixed biochemical constant. Rather, it represents the minimal fraction of cleaved SNARE molecules required to reduce the probability of vesicle fusion below effective transmission levels. The value of θ is expected to vary between synapse types, tissue architectures, disease states, and compensatory plasticity conditions, and should be treated as a distributed parameter subject to empirical estimation.

Here, the term field denotes a spatially indexed probability mapping and does not imply a physical or diffusive process.

The MPF is a probabilistic abstraction linking discrete molecular events to spatially distributed functional thresholds, distinct from pharmacokinetic or pharmacodynamic concentration models. It captures the likelihood that a given presynaptic terminal reaches a molecular state sufficient to alter neurotransmitter release. In this framework, clinical efficacy is determined not by the presence of toxin per se, but by the structure and extent of the probability field generated by its interaction with neural tissue.

### 4.2. Elementary Variables of the MPF

The MPF is governed by a finite set of elementary variables, each grounded in molecular or cellular biology. Concretely, the MPF can be understood as a probability mapping driven by (i) local exposure C(x,t) and exposure time, (ii) presynaptic terminal density, (iii) receptor-mediated binding and internalization probability, (iv) intracellular catalytic persistence/efficiency, and (v) a functional silencing threshold θ (with potential higher-level integration thresholds at the motor-unit level). This makes explicit that formulation-, tissue-, and technique-dependent factors enter the framework as probabilistic modulators of the same generative architecture.

First, the local concentration of botulinum toxin determines the availability of toxin molecules capable of engaging presynaptic receptors and entering nerve terminals [3]. However, concentration alone is insufficient to predict the outcome, as receptor-mediated uptake imposes a nonlinear relationship between extracellular presence and intracellular action.

Second, exposure time modulates the probability of productive interactions between toxin molecules and presynaptic terminals. Prolonged exposure increases the likelihood of internalization and subsequent enzymatic activity, even at lower concentrations.

Third, the density of presynaptic terminals within a given tissue volume defines the number of potential molecular targets. Anatomical variability in terminal density therefore directly shapes the spatial structure of the MPF and contributes to inter-individual differences in response [4].

Fourth, the probability of internalization reflects the expression of the receptor, the dynamics of the membrane, and the endocytic efficiency at the terminal level. This variable introduces intrinsic biological variability independent of the dose or technique injected.

Fifth, the probability of SNAP-25 cleavage depends on the catalytic efficiency of the toxin light chain once internalized. Serotype-specific differences in substrate recognition and cleavage kinetics further modulate this probability [7].

Each elementary variable is formally bounded by biological constraints. Internalization probability is defined on [0, 1]; terminal density corresponds to anatomically measurable distributions; the variability parameter σ represents dispersion of terminal-level probabilities and is non-negative; and exposure C(x,t) follows physical concentration constraints. Precise empirical ranges require dedicated measurement and are intentionally not imposed a priori to avoid artificial calibration. In a patient-level formulation, elementary variables such as terminal density, receptor expression, and compensatory plasticity can be represented as distributed random variables rather than fixed constants. Under this view, $P_{\mathrm{MPF}}\left(x,t\right)$ becomes a conditional stochastic field defined over parameter distributions, enabling population-level variability modeling without altering the core probabilistic architecture.

Finally, a molecular threshold of functional silencing must be exceeded for neurotransmission to be significantly impaired. Below this threshold, partial cleavage events may occur without producing a detectable functional effect. This threshold property transforms a continuum of molecular events into a discrete functional outcome. Together, these variables define the MPF as a molecularly grounded probabilistic object rather than a deterministic field.

### 4.3. From Local Probability to Emergent Functional Effect

The functional effect of botulinum toxin emerges from the spatial integration of molecular cleavage events across populations of presynaptic terminals. Individual terminals contribute discretely to neurotransmission; only when a sufficient proportion within a functional unit is silenced does a measurable reduction in muscle activation or neurotransmitter release occur.

At the motor unit level, this integration gives rise to a second threshold: a critical fraction of affected terminals must be reached to exceed functional redundancy and produce observable weakness or modulation of activity [6]. Consequently, identical molecular events at the single-terminal level can yield divergent functional outcomes depending on their spatial distribution and density.

This framework explains how gradual changes in molecular probability—driven by concentration, exposure time, or injection geometry—can result in abrupt transitions in functional effect. It also clarifies why small variations in technique or anatomy may lead to disproportionate differences in clinical outcome, even in the presence of an invariant molecular mechanism.

By explicitly linking discrete molecular events with emergent tissue-level behavior, the MPF-BoNT framework provides a principled bridge between molecular biology and functional physiology. The multilevel structure of the MPF-BoNT framework, which spans the molecular, cellular, spatial, and system levels, is summarized in Table 1.

### Relationship to Classical PK/PD Frameworks

The MPF-BoNT framework does not replace pharmacokinetic (PK) or pharmacodynamic (PD) models but operates at a complementary level. PK models describe concentration dynamics across tissue compartments, while PD models relate concentration to functional effect magnitude. In contrast, the MPF introduces an explicitly spatial–probabilistic object describing the likelihood that presynaptic terminals exceed a functional silencing threshold. Thus, PK primarily constrains exposure, PD relates exposure to magnitude, whereas MPF formalizes the spatial probability structure linking molecular events to functional thresholds.

## 5. Spatial Structure of the MPF: Integrating Diffusion and Probabilistic Threshold Dynamics

Diffusion-based descriptions remain essential for understanding local concentration gradients following injection. The present framework does not negate diffusion processes but situates them within a broader probabilistic interpretation that incorporates receptor-mediated uptake, terminal heterogeneity, and functional emergence. In this sense, diffusion constitutes one component of exposure within the MPF architecture rather than a competing explanatory paradigm.

### 5.1. Spread as the Tail of a Probability Distribution

In clinical and experimental discourse, the term “spread” is often used to describe botulinum toxin effects observed beyond the intended target area. Such effects are frequently interpreted as technical errors or unwanted diffusion. However, within the Molecular Probability Field (MPF) framework, spread can be interpreted as an intrinsic statistical property of the field itself rather than solely as a technical diffusion error.

Importantly, the presence of a probabilistic tail is unavoidable whenever molecular interactions occur across heterogeneous tissue environments. Terminals located on the periphery of the injection site may still reach the molecular threshold for functional silencing, although with a lower probability. When integrated across space, these low-probability events can yield detectable functional consequences, particularly in anatomically dense or functionally sensitive regions [4,8].

Thus, within the MPF framework, spread is a predictable statistical outcome arising from the spatial structure of molecular probability, not a violation of mechanistic specificity.

### 5.2. Injection Pattern, Concentration, and Spatial Anisotropy

The spatial configuration of the MPF is strongly influenced by injection-related parameters that shape local molecular exposure. Moreover, identical total doses do not generate identical MPFs. Variations in injection pattern, aliquot size, and spatial geometry alter the distribution of local concentrations and exposure times, thus reshaping the probability field.

For example, a single bolus injection produces a steep concentration gradient with a narrow region of high probability surrounded by a relatively extended low-probability tail. In contrast, multiple smaller aliquots distributed across a target region generate overlapping probability fields that can produce a broader, more homogeneous MPF. These differences arise despite identical total doses and reflect spatial anisotropy rather than changes in molecular mechanism.

This anisotropic structure is further modulated by tissue architecture and presynaptic terminal density, which influence how local molecular probabilities are translated into functional effects [3]. Consequently, injection geometry acts as a higher-order determinant of the MPF, governing both efficacy and the likelihood of peripheral effects.

By reframing injection parameters as modulators of spatial probability rather than diffusion drivers, the MPF model provides a principled explanation for why similar doses can produce divergent clinical outcomes. It also clarifies how deliberate manipulation of injection geometry can be used to sculpt the probability field, optimizing target engagement while minimizing unintended effects.

## 6. Temporal Evolution of the MPF: Why Effects Wear off

### 6.1. Decline in Effect as System Reorganization, Not Toxin Disappearance

The temporal decline in botulinum toxin effects is often described clinically as a gradual loss of efficacy over time. However, within the Molecular Probability Field (MPF) framework, this decline is not attributed to the disappearance or inactivation of the toxin itself, but rather to multilevel biological reorganization following the initial molecular insult.

At the molecular level, the persistence of the activity of the botulinum toxin light chain and the turnover of cleaved SNAP-25 molecules define the duration of effective synaptic silencing [1,2]. As newly synthesized SNARE proteins replace cleaved substrates, the probability of sustained neurotransmission blockade progressively decreases, contributing to the temporal contraction of the MPF.

At the cellular level, compensatory synaptic mechanisms further modulate recovery.

The experimental literature supports that synaptic terminals can exhibit compensatory structural and functional responses to sustained blockade (e.g., remodeling and altered organization of the release site); however, the extent to which specific compensatory mechanisms dominate between tissues and indications likely varies and remains an empirical question [6]. These adaptive responses do not negate the molecular action of the toxin but reduce its functional impact by reorganizing synaptic connectivity.

At the system level, neuromuscular recalibration contributes to the observed decline in effect. Motor units adapt to altered patterns of synaptic input, redistributing load across unaffected fibers, and adjusting recruitment strategies. This functional plasticity further decouples the persistence of molecular cleavage from the sustained clinical effect, reinforcing the view that recovery reflects system-level adaptation rather than molecular failure [4].

### 6.2. MPF Dynamics over Time

The MPF evolves dynamically after toxin administration, exhibiting characteristic temporal phases that reflect the underlying molecular and cellular processes.

The initial phase of MPF establishment is dominated by increasing probabilities of terminal internalization and SNAP-25 cleavage as toxin exposure accumulates. During this phase, the spatial extent and intensity of the probability field expand until a critical proportion of terminals within functional units is silenced.

This is followed by a plateau phase, during which the MPF remains relatively stable. Molecular cleavage events have reached near-saturation within high-probability regions, and functional effects are maintained despite ongoing biological turnover. The duration of this plateau varies between tissues and individuals, reflecting differences in synaptic density, compensatory capacity, and molecular turnover rates [3].

Finally, the regression phase is characterized by a gradual contraction of the effective MPF. As synaptic repair, protein turnover, and functional reorganization proceed, the probability that the terminals remain functionally silenced falls below the threshold required for the observable effect. Importantly, this regression does not occur uniformly across space; the peripheral regions of the MPF regress earlier than the central regions, resulting in a shrinking but not abrupt loss of functional impact.

By explicitly modeling these temporal dynamics, the MPF framework accounts for the characteristic onset, persistence, and recovery patterns observed with botulinum toxin, without invoking changes in toxin identity or mechanism.

## 7. Clinical and Experimental Implications

### 7.1. Testable Predictions

A central strength of the Molecular Probability Field (MPF) framework lies in its ability to generate explicit, falsifiable predictions that link molecular events to functional outcomes.

First, the model predicts that identical total doses of botulinum toxin administered using different injection patterns will generate distinct MPFs. Variations in aliquot size, spatial distribution, and injection geometry alter local concentrations and exposure times, thereby reshaping the spatial probability distribution of SNAP-25 cleavage. Consequently, equivalent doses can produce different efficacy profiles and patterns of peripheral effects, even in the absence of differences in toxin identity or molecular mechanism [3,8].

Second, the MPF framework predicts that comparable initial probability fields may nevertheless exhibit divergent temporal trajectories. Two tissues with similar initial MPFs may show different durations of effect due to differences in synaptic compensation, protein turnover, and neuromuscular recalibration. This prediction follows directly from the separation between molecular cleavage events and the system-level reorganization processes described above [6].

Third, the model provides a principled basis for distinguishing technical non-response from biological non-response. A technical non-response arises when the generated MPF fails to reach the molecular threshold required for functional silencing, due to factors such as inadequate targeting or unfavorable spatial distribution. In contrast, a biological non-response reflects reduced internalization, altered cleavage probability, or enhanced compensatory mechanisms at the terminal or system level. This distinction has direct implications for the experimental interpretation and for the mechanistically grounded interpretation of heterogeneous response patterns in applied settings [4].

In practical terms, a geometry-limited pattern motivates hypotheses about targeting and spatial redistribution (e.g., field shape sculpting), whereas a biology-limited pattern motivates hypotheses about altered uptake/cleavage competence, threshold variability, or compensation. This distinction is not a treatment recommendation, but a structured way to decide which variables should be interrogated next when outcomes diverge under comparable nominal dosing. A concrete experimental falsification strategy follows directly from the MPF formulation. For example, a controlled design in which identical nominal doses are administered using systematically varied injection geometries (e.g., single bolus versus distributed micro-aliquots), combined with spatial mapping of cleaved SNARE fragments or terminal-level transmission failure, would test whether distinct spatial probability structures emerge despite equal total exposure. If no measurable divergence in spatial–functional profiles were observed under such controlled variation, the core spatial thesis of the MPF would be weakened. Conversely, reproducible spatial divergence under controlled geometry would provide empirical support for the probabilistic field interpretation.

Within this framework, MPF is not itself an observable quantity, but a generative construct whose integrals or level-set statistics can be related, in experimental settings, to measurable proxies such as the fraction of SNAP-25 cleaved terminals, the spatial extent of neuromuscular transmission failure, or the probability distribution of functional suppression across motor units.

### 7.2. Implications for Experimental Design

Clinician-facing interpretive outputs. Although MPF-BoNT does not provide dosing rules or procedural prescriptions, it constrains how spatial–functional divergence should be interpreted under fixed nominal dose conditions. In particular, the framework implies that (i) geometry is a primary determinant of spatial–functional outcome at fixed nominal dose; (ii) “spread” should be interpreted as a tail-risk property of the probability field rather than as a categorical technical error; and (iii) apparent non-response should be parsed into geometry-limited vs. biology-limited regimes, prompting different next-step hypotheses (retargeting/redistribution vs. altered uptake/threshold/compensation). These outputs are intended as qualitative decision checks that can be operationalized only after calibration, but they already constrain interpretation of heterogeneous outcomes within a mechanistically anchored language.

The MPF framework also informs the design of experimental strategies aimed at elucidating the action of botulinum toxin beyond descriptive outcome measures. At the molecular level, imaging approaches capable of visualizing the cleavage of SNAP-25 or the presence of cleaved SNARE fragments provide a direct readout of local molecular events. Such methods enable spatial mapping of molecular probability fields and facilitate the correlation between cleavage distributions and functional effects [1,2].

At the cellular level, proxy markers, such as changes in synaptic density, dynamics of vesicle recycling, or alterations in the probability of neurotransmitter release, can serve as indirect indicators of MPF structure and evolution over time. These measures allow integration of molecular events with cellular adaptation processes [6].

Finally, animal models and computational simulations offer complementary platforms for testing MPF-based predictions. Controlled manipulation of injection geometry, concentration, and exposure time in vivo can be combined with local pharmacokinetic simulations to explore how molecular probability fields arise and regress within anatomically realistic environments [4].

A minimal validation protocol would require: (i) controlled manipulation of injection geometry at equal nominal dose; (ii) spatially resolved measurement of cleaved SNARE fragments or transmission failure; (iii) temporal tracking of field contraction dynamics; (iv) comparison between predicted and observed spatial divergence patterns. Failure to detect controlled spatial divergence under controlled conditions would challenge the spatial component of the current MPF formulation.

Together, these implications illustrate how the MPF framework functions not merely as a conceptual abstraction but as a practical guide for hypothesis generation, experimental design, and interpretation across molecular, cellular, and systems levels. A minimal numerical illustration of the MPF-BoNT framework, designed to visualize its spatial and threshold-based behavior without empirical fitting, is provided in the Supplementary Material S2.

### Pathway Toward Quantitative Instantiation

Although the present manuscript does not introduce calibrated parameter values, the MPF framework is amenable to numerical implementation once terminal-level measurements become available. Empirical estimation of internalization probabilities, catalytic persistence, presynaptic density distributions, and functional suppression thresholds would permit simulation-based instantiation of MPF(x,t) and sensitivity analysis of spatial–temporal dynamics. Such calibration would transform the current structural framework into a predictive computational model, a step intentionally reserved for future empirical work. In practical terms, this would enable prospective comparison of injection geometries and spatial response patterns within a unified probabilistic metric.

## 8. Scope, Limits, and Non-Claims

The Molecular Probability Field (MPF-BoNT) framework is intentionally framed as a conceptual and theoretical construct designed to organize existing molecular knowledge and generate testable hypotheses. As such, its scope and limitations must be clearly delineated.

First, the MPF model does not introduce new molecular mechanisms of botulinum toxin action. Enzymatic cleavage of SNAP-25 and the associated inhibition of neurotransmitter release remain the only molecular events considered, consistent with well-established biochemical evidence [1,2]. The model reinterprets how these known events are distributed and integrated across space and time, without proposing additional pathways or targets. Consequently, the MPF-BoNT framework is not intended to describe pathological conditions involving axonal degeneration, altered axonal transport, or non-canonical toxin uptake pathways, which fall outside the scope of the present formulation.

Second, the MPF framework does not make claims about direct central nervous system effects of peripherally administered botulinum toxin. Any discussion of central modulation is explicitly limited to indirect consequences of peripheral synaptic reorganization and altered afferent signaling, in line with existing cautious interpretations in the literature [9]. Differences among botulinum neurotoxin serotypes (e.g., molecular targets, cleavage efficiency, duration of action, and clinical dose) are not modeled as distinct mechanisms, but are incorporated parametrically within the MPF framework, through their effects on threshold, variability, spatial organization, and temporal dynamics.

Importantly, this parametric stance also accommodates contemporary engineered or recombinant toxins. Variants optimized for receptor binding, tissue retention/spread, catalytic persistence, or clinical duration do not invalidate the MPF concept; they correspond to systematic shifts in the elementary probability determinants (e.g., binding/internalization, spatial tails, plateau length, or regression dynamics). The key point is that such innovations can be represented as parameter modulations of the same probabilistic field rather than requiring a separate explanatory model.

For clarity, Table 2 summarizes how well-established molecular and clinical differences among botulinum neurotoxin serotypes are expressed parametrically within the MPF-BoNT framework, without introducing toxin-specific mechanisms or model structures.The model therefore avoids speculative assertions that extend beyond current molecular evidence.

Parametric representation of serotype differences does not imply biological equivalence. Rather, it reflects a structural modeling choice in which distinct intracellular persistence profiles, recovery kinetics, and spatial spread characteristics are encoded as parameter shifts within a shared probabilistic architecture. Should future evidence demonstrate qualitatively distinct intracellular dynamical regimes, the MPF framework could accommodate serotype-specific dynamical kernels without altering its foundational structure.

Third, the MPF model does not compare commercial formulations, serotypes, or units of botulinum toxin, nor does it assert equivalence or superiority among products. Differences in formulation, potency units, or manufacturing processes are acknowledged only insofar as they may influence elementary molecular variables such as concentration or internalization probability, without serving as objects of comparison.

The present formulation intentionally focuses on peripheral neuromuscular synapses, where terminal-level molecular events are directly measurable and spatially localizable. Extension to central nervous system contexts would require incorporation of retrograde transport dynamics, network-level plasticity, and multi-synaptic integration, which fall beyond the methodological scope of this initial instantiation.

Finally, MPF-BoNT should be understood as a generative framework rather than a predictive algorithm. It is intended to formalize existing observations, clarify sources of variability, and guide experimental design by articulating explicit, falsifiable predictions. Empirical validation and quantitative refinement of the model remain essential future steps and lie beyond the scope of the present work. Within its intended scope, the MPF-BoNT framework is primarily designed to support mechanistic study design and interpretation by clarifying which molecular, spatial, and functional variables should be measured, at which level of organization, and how technical sources of non-response may be distinguished from biological variability.

By explicitly defining these boundaries, the MPF framework aims to provide conceptual clarity while avoiding overextension beyond what current molecular and experimental evidence can support.

## 9. Conclusions

In this work, we introduce the Molecular Probability Field (MPF-BoNT) as a formal framework that links molecular cleavage events to observable spatial, temporal, and functional phenomena.

By linking discrete molecular cleavage events to emergent spatial–temporal functional patterns, the MPF-BoNT framework provides a structurally explicit language for interpreting variability in botulinum toxin effects across molecular, cellular, and systems levels.

For clinicians and experimental researchers, the central interpretive implication is the following: identical nominal doses can yield distinct spatial–functional outcomes because functional effects emerge from spatial integration of probabilistic terminal silencing. The MPF framework provides a mechanistically anchored language to describe and test this source of variability without implying prescriptive dosing rules.

## Figures and Tables

**Table 1:** The table summarizes how discrete molecular events induced by botulinum toxin are integrated across hierarchical biological levels to generate emergent functional effects. At the molecular level, SNAP-25 cleavage represents the elementary enzymatic event. At higher levels, probabilistic terminal silencing, spatial organization of molecular probabilities, and threshold-dependent integration within functional units collectively determine tissue-level efficacy, spatial spread, and temporal evolution of botulinum toxin effects.

Level	Primary Process	Description
Molecular	SNAP-25 cleavage	Discrete enzymatic cleavage of SNAP-25 by the botulinum toxin light chain, constituting the fundamental molecular event underlying synaptic transmission blockade.
Cellular	Terminal silencing probability	Probability that an individual presynaptic terminal undergoes sufficient molecular cleavage to exceed the functional silencing threshold.
Spatial	Molecular Probability Field (MPF)	Spatial–temporal distribution of silencing probabilities across tissue, shaped by local toxin concentration, exposure time, presynaptic terminal density, and internalization efficiency.
Functional unit	Threshold integration	Integration of molecular probabilities across populations of presynaptic terminals within a motor unit or neural circuit, giving rise to nonlinear, threshold-dependent functional effects.
Systems	Adaptive reorganization	Temporal evolution of functional outcomes driven by molecular turnover, synaptic compensation, and neuromuscular recalibration rather than toxin disappearance.

**Table 2:** This table illustrates how established biological, clinical, and engineered differences among botulinum neurotoxin serotypes or formulations are incorporated within the MPF-BoNT framework through parameter modulation rather than through toxin-specific mechanisms or distinct model structures. Receptor binding, catalytic efficiency, intracellular persistence, spatial spread, and dosing effects are represented as shifts in the probabilistic determinants shaping the Molecular Probability Field. This preserves a unified generative architecture while allowing serotype- and formulation-dependent heterogeneity to be represented quantitatively. For simplicity, the main text writes $C_{\mathrm{SNAP}25}$ for BoNT/A; in the serotype-general formulation, the same event is denoted $C_{\mathrm{SNARE}}$ to indicate the appropriate substrate (e.g., SNAP-25, VAMP, syntaxin).

Serotype/Formulation Specific Feature	Representation
Different SNARE target (e.g., SNAP-25 vs. VAMP)	Definition of the molecular event variable $C_{\mathrm{SNARE}}$
Different catalytic cleavage efficiency	Molecular activation threshold θ and local cleavage probability term
Enhanced receptor binding affinity or altered receptor selectivity	Internalization probability term; effective uptake nonlinearity
Altered tissue retention or spread characteristics	Spatial structure of the MPF (anisotropy; modulation of probabilistic tails)
Engineered prolonged intracellular persistence	Temporal dynamics of the MPF (plateau duration; regression rate)
Different clinical duration of action	Contraction/expansion rate of the effective MPF over time
Different clinical dosing regimens	Scaling of local exposure C(x,t)
Different inter-terminal variability	Variability parameter σ governing dispersion of terminal-level probabilities

## Data Availability

No new data were created or analyzed in this study. Data sharing is not applicable to this article.

## Supplementary Materials

The following supporting information can be downloaded at: https://www.mdpi.com/article/10.3390/biology15050446/s1. Supplementary Material S1. Mathematical Formalization of the Molecular Probability Field (MPF-BoNT). This document provides the full probabilistic derivation of the discrete stochastic framework, including Bernoulli terminal activation modeling, collective threshold behavior, spatial geometry dependence, and analytical comparison with continuous mean-field (Hill-type) formulations. Supplementary Material S2. A Minimal Numerical Illustration of the MPF-BoNT Framework. This document presents a numerical example and simulation-based illustration of the MPF-BoNT model, demonstrating discrete threshold emergence, geometry-driven variability, and divergence from mean-field predictions under identical average concentration conditions.
