# Supplementary material for: From Molecular Cleavage to Clinical Effect: A Probabilistic Field Model of Botulinum Toxin Action
Source: Biology (Basel). 2026 Mar 9;15(5):446. doi: 10.3390/biology15050446 (PMC12984648; doi:10.3390/biology15050446)
Supplement: Supplementary file 1 [file biology-15-00446-s001.zip › S1_clean revised.pdf]

Supplementary Material S1

S1 Mathematical Formalization of the Molecular Probability Field (MPF-BoNT)

This Supplementary Material provides a formal mathematical description of the Molecular Probability Field (MPF-BoNT) introduced in the main text. The purpose of this formalization is not to define a fully parameterized predictive model, but to establish a rigorous conceptual structure that links discrete molecular events to emergent tissue-level effects in a probabilistic and spatial-temporal framework.

S1.1 Elementary Molecular Event

At the most fundamental level, the action of botulinum toxin is represented by a binary molecular event occurring at individual presynaptic terminals. Let $C_{\mathrm{SNAP25}}(x,t)$ denote the local extent of SNAP-25 cleavage at spatial location x and time t. Functional silencing of neurotransmission occurs when this quantity exceeds a molecular threshold θ, corresponding to sufficient impairment of SNARE-mediated vesicle fusion.

Let

$$F(x,t) \equiv \{C_{\mathrm{SNAP25}}(x,t) \geq \theta\} \tag{S1}$$

denote the terminal-level functional event. The Molecular Probability Field is therefore defined as

$$\mathrm{MPF}(x,t) = P(F(x,t))\,. \tag{S2}$$

Here $P(\cdot)$ denotes probability. This formulation explicitly distinguishes the deterministic nature of the enzymatic cleavage reaction from the probabilistic nature of its occurrence across a heterogeneous population of terminals.

Importantly, although the formal definition is expressed in terms of SNAP-25 cleavage, the probability $P(F(x,t))$ implicitly depends on upstream determinants including exposure, receptor-mediated internalization, catalytic persistence, and terminal density, consistent with the integrative structure described in the main manuscript.

S1.2 Probabilistic Decomposition of the MPF

To avoid implicit independence assumptions, we express the MPF via a chain of conditional events at the terminal level. Let I denote the event that a toxin molecule (or effective toxin unit) is internalized by a presynaptic terminal, and let K denote the event that an internalized light chain remains catalytically competent over the relevant time window.

$$\mathrm{MPF}(x,t) = P(F(x,t)) = \sum_{i\in\{0,1\}} \sum_{k\in\{0,1\}} P(F \mid I=i, K=k)\, P(K=k \mid I=i)\, P(I=i \mid x,t). \tag{S3}$$

All conditional probabilities are defined on the interval $[0,1]$, and no parametric distributional form is imposed at this stage. In many plausible biological regimes, $P(F \mid I=0, K=k) \approx 0$ because cleavage requires internalization. Likewise, if $K=0$ denotes loss of catalytic competence, then $P(F \mid I=1, K=0)$ is expected to be low. Importantly, the formulation above makes no factorization assumptions; it simply states that the probability of functional silencing can be decomposed into interpretable conditional components that correspond to known molecular steps.

S1.3 Spatial Integration and Emergent Functional Effect

While the molecular event is defined at the level of individual presynaptic terminals, functional outcomes emerge at higher organizational levels. Let Ω denote the anatomical domain of interest, and let $\rho(x)$ represent the local density of presynaptic terminals.

The aggregate functional effect at time t can be represented as

$$E(t) = \int_{\Omega} \mathrm{MPF}(x,t)\, \rho(x)\, dx. \tag{S4}$$

If $\rho(x)$ is interpreted as a terminal density (e.g., terminals per unit tissue volume), then $E(t)$ has the meaning of an expected number (or expected mass) of functionally silenced terminals within Ω at time t. Depending on the experimental readout, $E(t)$ can be mapped to an observable proxy (e.g., reduction in evoked release probability) via a monotone link function, without changing the MPF definition.

This expression formalizes the concept that measurable changes in neurotransmission or muscle activation arise only when a sufficient fraction of terminals within a functional unit is silenced. It also provides a principled explanation for threshold-like behavior, whereby gradual changes in molecular probability produce abrupt functional transitions.

S1.4 Thresholds and Nonlinearity

Two distinct thresholds are implicit in the MPF-BoNT framework. The first is the molecular threshold θ, which governs functional silencing at the single-terminal level. The second is a system-level threshold that reflects redundancy within motor units or neural circuits.

The parameter θ should not be interpreted as a fixed biochemical constant. Rather, it represents a distributed functional criterion reflecting the minimal fraction of cleaved SNARE complexes required to impair effective neurotransmission. Its value is expected to vary across synapse types, tissue architectures, and disease states.

Nonlinearity arises naturally from the interaction between these thresholds and the spatial distribution of molecular probabilities. As a consequence, dose escalation or changes in injection geometry may produce disproportionate effects without invoking nonlinear molecular kinetics.

S1.5 Temporal Dynamics of the MPF

The temporal evolution of the MPF reflects the balance between molecular and adaptive processes. In qualitative form, the dynamics of the probability field may be expressed as

$$\Delta \mathrm{MPF}(x,t) \propto +f_{\mathrm{uptake}}(x,t) - f_{\mathrm{turnover}}(x,t) - f_{\mathrm{compensation}}(x,t). \quad \text{(S5)}$$

The term f_{uptake} represents processes increasing the probability of SNAP-25 cleavage, such as ongoing internalization. The term f_{turnover} represents molecular recovery driven by SNARE protein resynthesis. The term $f_{\mathrm{compensation}}$ represents synaptic and system-level adaptive mechanisms.

This expression is a qualitative bookkeeping identity that partitions contributions to MPF change over time; it is not specified as a closed-form dynamical system without additional empirical assumptions.

S1.6 Scope of the Mathematical Framework

The mathematical expressions presented here serve to formalize the logical structure of the MPF-BoNT model. They define relationships between molecular events, spatial organization, and functional outcomes without requiring parameter estimation or empirical calibration.

As such, the framework is generative rather than predictive. Its primary function is to clarify assumptions, guide experimental design, and provide a coherent language for interpreting variability in botulinum toxin effects across molecular, cellular, and systems levels. Issues of parameter identifiability are therefore not addressed here, as the MPF is formulated as a conceptual mapping rather than an inferential or data-fitting model.
