# Supplementary material for: From Molecular Cleavage to Clinical Effect: A Probabilistic Field Model of Botulinum Toxin Action
Source: Biology (Basel). 2026 Mar 9;15(5):446. doi: 10.3390/biology15050446 (PMC12984648; doi:10.3390/biology15050446)
Supplement: Supplementary file 1 [file biology-15-00446-s001.zip › S2-clean revised.pdf]

Supplementary Material S2

S1 A Minimal Numerical Illustration of the MPF-BoNT Framework

This supplementary material provides a simple numerical illustration of the Molecular Probability Field (MPF-BoNT) framework. The purpose of this illustration is not quantitative prediction or empirical validation, but visualization of how probabilistic molecular events, spatial organization, and threshold effects interact within the proposed framework.

S1.1 General Setup

We consider a one-dimensional tissue domain $\Omega = [0, L]$, representing a simplified segment of muscle or neural tissue. Presynaptic terminals are assumed to be uniformly distributed with constant density $\rho(x) = \rho_0$. All quantities are normalized and dimensionless.

Local toxin exposure is represented by a spatial concentration profile $C(x)$, which differs between injection patterns, but integrates into the same total dose in Ω.

The Molecular Probability Field is defined as

$$\mathrm{MPF}(x) = \Phi\left(\frac{C(x) - \theta}{\sigma}\right) \tag{S1}$$

where Φ denotes any monotone mapping from exposure to probability (e.g., a CDF-like sigmoid) used solely for visualization purposes; σ is an illustrative dispersion parameter controlling transition sharpness and does not represent an empirically estimated variance in the present manuscript; θ is the molecular threshold for functional silencing. For simplicity, time dependence is suppressed in this illustrative example.

The aggregate functional effect is given by

$$E = \int_\Omega \mathrm{MPF}(x)\, \rho_0 \, dx. \tag{S2}$$

The specific choice of Φ is illustrative; any monotonic mapping from exposure to probability would preserve the qualitative behavior. The apparent simplicity of the numerical mapping is intentional: the illustrative

power of the example lies not in the functional form itself but in the separation between local molecular certainty and aggregate probabilistic effect, which persists under any monotone probability mapping.

Example 1: Single Bolus Injection

In the first example, toxin exposure is modeled as a single localized bolus centered at x_0:

$$C_1(x) = A \exp\left(-\frac{(x - x_0)^2}{2w^2}\right) \tag{S3}$$

where A controls the peak concentration and w controls the spatial spread.

This configuration produces a narrow MPF with a high central probability and extended low-probability tails. Functional silencing is dominated by a compact region where $C_1(x)$ exceeds the threshold θ.

Example 2: Multiple Distributed Micro-Aliquots

In the second example, the same total dose is distributed across four micro-aliquots:

$$C_2(x) = \sum_{k=1}^{4} \frac{A}{4} \exp\left(-\frac{(x - x_k)^2}{2w^2}\right) \tag{S4}$$

with x_k evenly spaced across Ω.

Although $\int_\Omega C_2(x)\,dx = \int_\Omega C_1(x)\,dx$, the resulting MPF is wider and more homogeneous. A larger fraction of the domain exceeds the threshold θ, yielding a larger effective E despite the identical total dose.

Example 3: Identical Initial MPF with Different Threshold Sensitivity

In the third example, the spatial concentration profile is kept constant, but the variability parameter σ is altered to represent different biological compensation regimes.

For smaller σ, the MPF exhibits a sharp transition around θ, resulting in abrupt functional onset and regression. For larger σ, the MPF transitions are smoother, leading to prolonged partial silencing and a slower decline of E over time.

This example illustrates how identical initial molecular exposure can produce different temporal and functional outcomes because of differences in terminal-level variability and system-level compensation.

S2.5 Interpretation

Together, these examples demonstrate that the MPF-BoNT framework naturally accounts for key qualitative phenomena described in the main text: non-equivalence of dose and effect, dependence on spatial injection pattern, threshold-driven nonlinearity, and variability in duration. Importantly, these behaviors emerge without the introduction of additional molecular mechanisms or empirical parameter fitting.
